# Social factors, wearable activity tracker use frequency, and physical activity patterns among U.S. older adults: findings from a national cross-sectional survey

**DOI:** 10.1186/s12877-026-07528-1

**Published:** 2026-04-23

**Authors:** Mengchi Li, Chakra Budhathoki, Hae-Ra Han, Sarah L. Szanton, Jennifer A. Schrack, Junxin Li

**Affiliations:** 1 School of Nursing, Health Science Center, Xian Jiaotong University, N0.76 Yanta West Rd, Xi’an, Shaanxi China; 2 School of Nursing, Johns Hopkins University, 525 N. Wolfe St, Baltimore, MD 21215 USA; 3 Bloomberg School of Public Health, Johns Hopkins University, 615 N. Wolfe St, Baltimore, MD 21215 USA

**Keywords:** Social factor, Wearable activity tracker, Older adult, Physical activity, Social determinants of health

## Abstract

**Background:**

Few studies have investigated wearable activity tracker (WAT) use frequency and physical activity (PA) patterns among older adults. The present study aims to describe PA patterns (moderate-to-vigorous physical activity (MVPA) and sedentary behavior) and to examine the association of social factors and WAT use frequency with PA patterns among U.S. older adults.

**Methods:**

We used cross-sectional self-reported data from 3,370 older adults from the Health Information National Trends Survey. Linear regression models and multinomial logistic regression models were used to identify associations among social factors, WAT use, and PA patterns. Mediation analysis was conducted to explore whether WAT use statistically explained the association between socioeconomic status and activity.

**Results:**

Most participants (63.7%) had < 150 min/week of MVPA, and 60.1% reported > 6 h/day of sedentary time. Female (b=-55.79, 95% CI: -101.5, -10.52) and older (≥ 75 years) adults (b=-51.47, 95% CI: -98.90, -4.04) had lower MVPA. Higher income was associated with higher MVPA (b = 60.90, 95% CI: 3.98, 117.81) and greater odds of “High MVPA & Low Sedentary” group (AOR = 2.16, 95% CI: 1.26, 3.68). College education was associated with higher odds of “High MVPA & Low Sedentary” group (AOR = 1.65, 95% CI: 1.08, 2.53). Frequent WAT use was associated with longer MVPA (b = 58.54, 95% CI: 4.73, 112.34) and “High MVPA & Low Sedentary” group (AOR = 1.92, 95% CI: 1.10, 3.34). Income had a significant indirect effect on MVPA through WAT use (IE = 4.08, 95% CI: 0.04, 8.13), but not education or sedentary behavior.

**Conclusion:**

Social disparities existed in PA patterns, but WAT use frequency partially explained the associations between income and MVPA time. This may inform a need to promote PA patterns especially in socially and economically disadvantaged older adults.

## Introduction

Physical activity (PA) is defined as any bodily movement requiring energy expenditure [30], and sedentary behavior is characterized by low-energy activities such as sitting [16]. Older adults should engage in moderate-to-vigorous physical activity (MVPA) for at least 150 min per week and reduce sedentary behavior to under 6 h per day for better health outcomes [5, 11, 30]. Unfortunately, the prevalence of inactivity significantly increases with age: an estimation of 26.9% of adults aged 65–74 years and 35.3% of adults aged 75 years and above were inactive [27]. Physical inactivity is strongly linked to mortality and morbidity such as diabetes, cardiovascular disease, cancer, obesity, mental illness, and dementia, threatening the health of the aging population [3]. Physical inactivity in the U.S. population results in approximately $117 billion in annual health care costs and about 10% of premature mortality [6]. There is an urgent need to improve PA among older adults to promote health, prevent chronic illnesses, and reduce health care costs in this population.

Studies show that older adults’ PA patterns may be influenced by multiple social factors. For example, older adults who are female, older, or have less education have been found to have lower PA levels on average [19]. Evidence on social factors associated with sedentary behavior in older adults have been mixed. Although some studies found that those who are males, older, with lower education (less than college) are more likely to engage in sedentary behavior in adults and older adults [8, 22], other evidence suggested no associations with sex, age, education, income and sedentary behavior in older adults [12]. These socioeconomic disparities in PA may be partially explained by differential access to resources that support active lifestyles. Access to innovative technologies such as wearable activity trackers (WATs)—consumer devices that provide feedback to the wearer such as fitness trackers, activity-tracking smartwatches, and pedometers [26]—has emerged as an additional determinant of PA among older adults. Given that WATs require financial investment, technological literacy, and smartphone compatibility, individuals with lower socioeconomic status may face greater barriers to WAT adoption, potentially exacerbating existing PA inequalities. Although previous evidence suggested that most U.S. older adults have never used a WAT [31], it has been reported that older adults show general interest and acceptance of using a WAT device to monitor their PA levels [15, 18]. WATs have demonstrated promising potential for PA enhancement in both observational and experimental studies with the aging population [33, 34].

Given the significant health and economic burden of physical inactivity and sedentary behavior among older adults, identifying modifiable determinants of PA patterns is essential. To the authors’ best knowledge, no study has simultaneously examined (1) how social factors are associated with older adults’ activity pattern groups—characterized by combined MVPA and sedentary time profiles—and (2) whether WAT use frequency, a potentially modifiable technology-facilitated behavior, modifies or mediates the relationship between socioeconomic status (SES) and PA patterns. While prior research has examined social factors in relation to MVPA or sedentary behavior separately, fewer studies have adopted a person-centered approach to classify older adults into distinct activity pattern groups. Furthermore, limited research has explored whether WAT use frequency (e.g., frequent vs. infrequent vs. non-use) is associated with PA patterns (Chandrasekaran et al., 2020), and no study has explicitly explored how WAT use explains the associations between SES and PA. Theoretically, SES influences PA partly through differential access to resources that enable active behaviors. WATs represent one such resource; however, they require financial investment for device purchase, technological literacy for operation, and smartphone compatibility for full functionality—resources that may be less accessible to older adults with lower SES [26]. Thus, WAT use may play a role on the SES-PA pathway by translating socioeconomic advantages into enhanced self-monitoring, motivation, and behavioral feedback. Understanding the interplay between social factors, WAT use, and PA patterns can inform targeted interventions to reduce health disparities and improve health outcomes among older adults.

The conceptual framework for the current study has been developed based on available evidence and adapted from the Fundamental Cause Theory (Link & Phelan, 1995). The Fundamental Cause Theory posits that SES can influence an individuals’ access to certain resources that may impact their health outcomes. In this study, WAT use was considered as an important technological resource that can influence health outcomes such as PA patterns. The purpose of this research study is to examine the associations among social factors, WAT use, and PA patterns among U.S. older adults. To address these objectives, this study pursued three specific aims:

- Aim 1: Examine social factors (e.g., sex, age, race and ethnicity, education, income) associated with older adults’ PA patterns (MVPA time- minutes per week, sedentary time -hours per day, and activity pattern groups). *Hypothesis*: Social factors are associated with older adults’ PA patterns.
- Aim 2: Examine the association between WAT use frequency and older adults’ physical activity. *Hypothesis*: Frequently WAT use is associated with higher MVPA time and lower sedentary time.
- Aim 3: Explore whether frequent use of WATs mediates the associations between socioeconomic status (income and education) and older adults’ PA patterns. *Hypothesis*: Frequent WAT use positively explains the association between socioeconomic status and PA patterns.

## Methods

### Study design and participants

This was a cross-sectional secondary data analysis using the Health Information National Trends Survey (HINTS) dataset with cross-sectional data collected from January to April 2019 and February to June 2020. Launched by the National Institutes of Health (National Cancer Institute) in 2003, HINTS regularly collects data about the American public’s knowledge of, attitudes toward, and use of cancer-related and other health-related information. To recruit participants, HINTS sent postal mail to random samples of non-vacant U.S. residential addresses for both the 2019 and 2020 cohorts. More details about study methods of the HINTS study are available through HINTS briefs and reports (Westat, [28], [29]). After excluded 9303 participants under 65 years of age from the total sample, the present study included 3,370 participants aged 65 and above from both 2019 and 2020 cohorts. All data collected in HINTS are self-reported on paper and sent back by mail.

### Ethics approval and consent

The HINTS 5 survey received expedited approval from the Westat Institutional Review Board (IRB #6048.14, March 28, 2016) and was classified as “Not Human Subjects Research” by the NIH Office of Human Subjects Research (Exempt #13204, April 25, 2016); informed consent was not required for primary data collection. The current secondary analysis of de-identified, publicly available HINTS 5 data was deemed non-human subjects research and exempt from further IRB review by the Johns Hopkins University School of Medicine IRB. Clinical trial registration: Not applicable.

### Measures

#### Social factors

Social factors included sex, age, race/ethnicity, annual household income, and education. Participants’ sex included “Male” and “Female.” Based on previous literature and data distribution, the age variable was dichotomized into 65–74 years and 75 years and above (Lee, Oh, Park, Choi, & Wee, 2018). Annual household income was categorized into (1) low income (less than $35,000), (2) intermediate income ($35,000-$75,000), and (3) high income ($75,000 or more) [31]. Education was categorized into (1) high school or less, (2) some college, and (3) college degree or higher. Race and ethnicity were categorized into three categories: (1) White, (2) Black, and (3) Hispanic, non-Hispanic Asians, and others.

#### WAT use

WAT use was assessed with two items: (1) “In the last 12 months, have you used a Wearable Activity Tracker to monitor or track your health or activity? (e.g., Fitbit, Apple Watch, Garmin Vivofit)” with response options “Yes” or “No”; and (2) “In the past month, how often did you use a wearable device to track your health?” with a 5-point frequency scale: “Every day,” “Almost every day,” “1–2 times per week,” “Less than once per week,” or “I did not use a wearable device in the past month.” Based on these items, WAT use was categorized as: (1) Frequent use —reported using a WAT “Every day” or “Almost every day”; (2) Infrequent use —reported using a WAT “1–2 times per week,” “Less than once per week,” or not in the past month but used one in the past year; and (3) No use —did not use a WAT in the past month or past 12 months.”

#### Physical activity patterns (PA Patterns)

While MVPA and sedentary behavior are distinct constructs, they are not mutually exclusive—individuals can meet activity guidelines yet still have sedentary lifestyles (Thivel et al., 2018). To capture this complexity, PA pattern was operationalized as a 4-category variable combining MVPA and sedentary time.

MVPA was assessed using two items: (1) “In a typical week, how many days do you do any physical activity of at least moderate intensity?” (0–7 days); and (2) “On those days, how long do you typically do these activities?” (minutes per day). The HINTS dataset provided a derived variable, “Minutes per week of at least moderate-intensity exercise,” which was used as the MVPA measure consistent with prior literature [31].

Sedentary time was assessed using one item: “During the past 7 days, how much time did you spend sitting on a typical day at home or at work?” (hours per day).

Based on current PA guidelines and literature, a weekly MVPA time under 150 min and a daily sedentary time more than 6 h were used as cutoffs for low MVPA time and high sedentary time (DHS, 2018). Four physical activity pattern groups were then created: (1) high MVPA low sedentary, (2) high MVPA high sedentary, (3) low MVPA low sedentary, and (4) low MVPA high sedentary groups.

#### Covariates

*Body Mass Index (BMI)* was calculated using the respondents’ self-reported heights and weights and was dichotomized into (1) non-obesity: under 30, and (2) obesity: 30 and above. *Smoking status* was a variable derived from two questions on past smoking experience and current smoking frequency and was categorized into current, former, and never smoker. The *marital status *question was included in the questionnaire and dichotomized into two categories: (1) married or living with a partner, and (2) divorced, widowed, separated, or never married. *Comorbidity *was measured by the sum of reported medical conditions reported by respondents and categorized into: (1) one or no comorbidity and (2) multiple comorbidities. *Mental health(depression and anxiety)* was assessed by the 4-item Patient Health Questionnaire-4 (PHQ-4) is an ultra-brief self-report validated questionnaire that consists of a 2-item depression subscale (PHQ-2) and a 2-item anxiety subscale (GAD-2) [20]. In each subscale, scores range from 0 to 6, with a score of 3 or greater indicating positive for depression or anxiety.

### Statistical analysis

Following the recommended methods, we first merged data from HINTS 5 cycle 3 and cycle 4, then accounted for the complex survey design using survey weights and the “delete one jackknife replication method” that deletes one sampling unit at a time from the full sample to create a set of 50 replicate weights (Westat, [28], [29]). The reported percentages are weighted and the sample sizes are unweighted. Analyses were conducted using Stata (version 17.0; StataCorp).

We first used descriptive analyses including frequency and percentages for categorical variables and means and standard deviations for continuous variables to summarize sample characteristics. We also used Pearson’s Chi-square to compare sample characteristics by PA patterns (weekly MVPA time, daily sedentary time, and activity pattern groups).

We then conducted multivariate linear regression analyses to identify social factors associated with older adults’ PA patterns. A test with a p-value of less than 0.05 was considered statistically significant. Multinomial logistic regression models were built to examine social factors associated with the activity pattern groups, with the low MVPA high sedentary group being the reference group. WAT use frequency associated with weekly MVPA time and daily sedentary time were examined using multiple linear regression models adjusting for covariates. WAT use frequency associated with activity pattern groups was assessed with a multinomial logistic regression model adjusting for covariates.

**Fig. 1:**
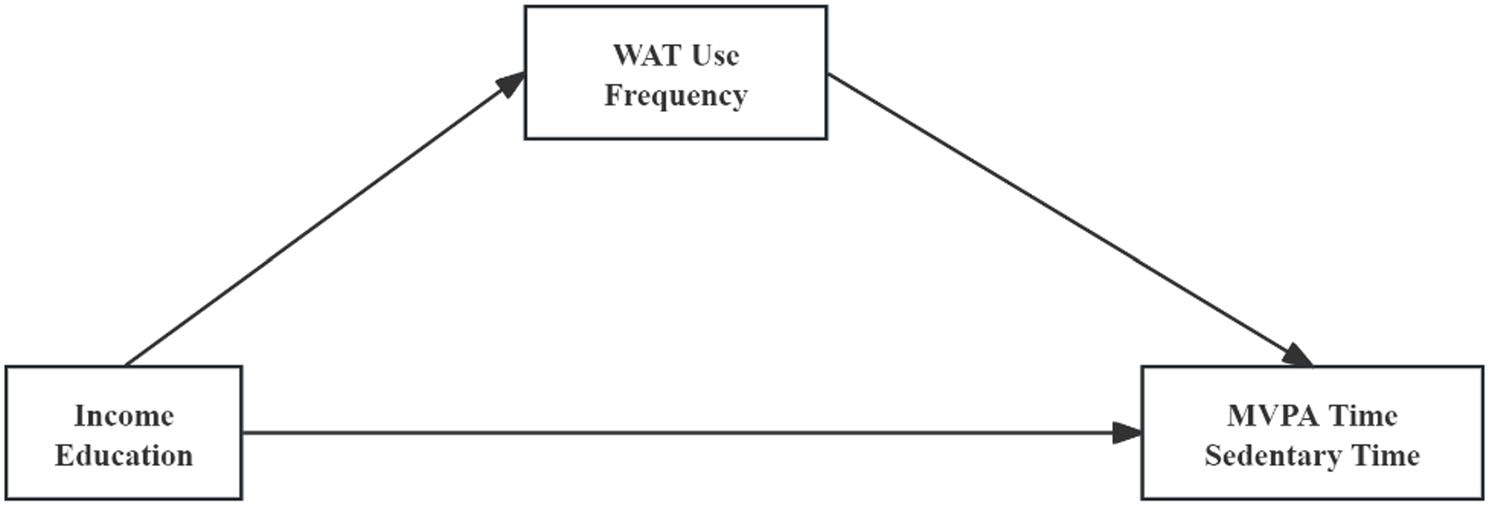
Mediation of WAT Use frequency on the association between SES and PA patterns

Finally, mediation analyses were performed to illustrate the association of SES (income and education) with physical activity levels (weekly MVPA minutes and daily sedentary hours) mediated by the use of WATs (Fig. 1). Baron and Kenny’s approach to test mediation (MEDSEM procedure in Stata version 17) was used to estimate the total effects, indirect effects (IE), and direct effects (DE) of SES on physical activity levels. Two models were estimated: a multivariate logistic regression model for WATs use (mediator) conditional on social factors (exposure), and all study confounders and a multivariate linear regression model for physical activity levels (outcome) conditional on social factors. The DE represented the effect of social factors on physical activity levels that were independent of WAT use. An IE represented the proportion of social factors that could be explained by its association with WATs use. Sobel Test was used to test the significance of IE. To quantify the magnitude of mediation, the study estimated the proportion of the association mediated by the use of WATs (IE/[DE + IE]).

## Results

### Sample characteristics by MVPA time, sedentary time, and activity pattern groups

Sample characteristics were described in total and by MVPA time, sedentary time, and activity pattern groups (Table 1). The current study included a total of 3370 participants, with 53.9% female, average age 73.9 years (SD = 7.1), 21.9% had a college degree or higher, and most were Non-Hispanic White (78.0%). Over half of the participants exercised less than 150 min per week (63.7%) and sat for more than 6 h per day (60.1%). The percentage of participants classified as the “Low MVPA and High Sedentary” class was 32.1%.

**Table 1:** Weighted sociodemographic and clinical characteristics by PA patterns

	Total	Weekly MVPA Time			Daily Sedentary Time			Activity pattern groups				
		< 150 min/wk	≥ 150 min/wk	*p*-value	< 6 h/day	≥ 6 h/day	*p*-value	Low MVPA High Sedentary	Low MVPA Low Sedentary	High MVPA High Sedentary	High MVPA Low Sedentary	*p*-value
	*N = *3370	*n* = 2,142 (63.7%)	*n* = 1,228 (36.3%)		*n* = 1,330 (39.9%)	*n* = 2040 (60.1%)		*n* = 1,086 (32.1%)	*n* = 1056 (31.6%)	*n* = 476 (13.9%)	*n =*752 (22.4%)	
Sex												
Male	1,374 (46.1%)	807 (58.8%)	567(41.2%)	**< 0.001**	515 (38.9%)	859 (61.1%)	0.341	411 (31.0%)	396 (27.7%)	229 (15.9%)	338 (25.4%)	**0.003**
Female	1,675 (53.9%)	1,142 (67.9%)	533 (32.1%)		683 (41.5%)	992 (58.5%)		569 (32.4%)	573 (35.5%)	194 (11.9%)	339 (20.2%)	
Age (years) 73.9 +- 1.7												
75&above	1,330 (41.6%)	912 (68.5%)	418 (31.5%)	**0.003**	491 (37.4%)	839 (62.6%)	0.116	473 (34.0%)	439 (34.5%)	180 (13.9%)	238 (17.6%)	**0.003**
65–74	2,040 (58.4%)	1,230 (60.4%)	810 (39.7%)		839 (41.7%)	1,201 (58.3%)		613 (30.8%)	617 (29.5%)	296 (13.9%)	514 (25.8%)	
Education												
High school degree and lower	1,024 (38.7%)	735 (70.1%)	289 (29.9%)	**< 0.001**	414 (40.0%)	610 (60.0%)	0.626	368 (35.8%)	367 (34.2%)	123 (12.3%)	166 (17.7%)	**<0.001**
Some college	1,036 (39.4%)	682 (62.9%)	354 (37.1%)		387 (39.0%)	649 (61.0%)		361 (32.4%)	321 (30.5%)	130 (13.6%)	224 (23.5%)	
College degree or higher	1,237 (21.9%)	683 (54.3%)	554(45.7%)		503 (42.1%)	734 (57.9%)		332 (24.7%)	351 (29.6%)	204 (15.8%)	350 (29.9%)	
Race and Ethnicity												
Non-Hispanic White	2,044 (78.0%)	1,280 (63.0%)	764 (37.0%)	0.135	760 (38.8%)	1,284(61.2%)	**0.005**	672 (33.4%)	608 (29.9%)	285 (13.2%)	479 (23.8%)	**<0.001**
Non-Hispanic Black	358 (9.2%)	255 (72.5%)	103 (27.5%)		149 (39.6%)	209 (60.4%)		121 (32.9%)	134 (39.7%)	43 (11.1%)	60 (16.3%)	
Hispanics, Asians, and others	486 (12.8%)	307 (64.4%)	179 (35.6%)		236 (50.8%)	250 (49.2%)		119 (20.6%)	188 (43.9%)	63 (13.9%)	116 (24.7%)	
Annual Household Income												
Less than 35k	1,150 (36.5%)	831(71.8%)	319 (28.2%)	**< 0.001**	411 (33.7%)	739 (66.3%)	**0.002**	448 (38.1%)	383 (33.7%)	145 (14.2%)	174 (14.0%)	**<0.001**
35k to less than 75k	964 (36.5%)	601 (63.4%)	363 (36.6%)		389 (41.4%)	575 (58.7%)		283 (30.7%)	318 (32.6%)	132 (11.8%)	231 (24.9%)	
75 K or more	765 (27.0%)	396 (51.7%)	366 (48.3%)		319 (46.1%)	443 (53.9%)		184 (22.7%)	212 (29.0%)	130 (16.2%)	236 (32.1%)	
Smoking Status												
Current Smoker	285 (8.6%)	203 (72.4%)	82 (27.6%)	**0.031**	110 (38.4%)	175 (61.6%)	**0.012**	101 (35.4%)	102 (37.0%)	34 (11.3%)	48 (16.3%)	**0.030**
Former Smoker	1,189 (37.2%)	775 (65.6%)	414 (34.4%)		420 (35.9%)	769 (64.1%)		430 (36.8%)	345 (28.8%)	148 (12.4%)	266 (22.0%)	
Never Smoker	1,835 (54.2%)	1,133 (61.6%)	702 (38.4%)		792 (43.7%)	1043 (56.3%)		535 (28.5%)	598 (33.1%)	267 (14.3%)	435 (24.2%)	
Body Mass Index (BMI)												
BMI under 30	2,345 (70.8%)	1,397 (60.3%)	948 (39.7%)	**< 0.001**	989 (43.4%)	1,356 (56.6%)	**< 0.001**	679 (28.9%)	718 (31.5%)	339 (14.3%)	609 (25.4%)	**<0.001**
BMI 30 or higher	1,025 (29.2%)	745 (72.0%)	280 (28.0%)		341 (31.5%)	684 (68.5%)		407 (40.1%)	338 (31.9%)	137 (12.9%)	143 (15.1%)	
Marital Status												
Married or living with a partner	1,559 (58.3%)	916 (60.9%)	643 (39.1%)	**0.003**	655 (41.9%)	904 (58.1%)	0.081	425 (29.4%)	491 (31.5%)	229 (13.7%)	414 (25.4%)	**0.003**
Divorced, Widowed, Separated, or never married	1,744 (41.7%)	1,191 (68.4%)	553 (31.6%)		649 (37.3%)	1095 (62.7%)		641 (36.0%)	550 (32.3%)	228 (13.1%)	325 (18.6%)	
Comorbidity												
One or No Comorbidity	1,777 (56.1%)	1,044 (57.8%)	733 (42.2%)	**< 0.001**	781 (45.0%)	996 (55.0%)	**< 0.001**	482 (26.7%)	562 (31.1%)	243 (13.5%)	490 (28.8%)	**<0.001**
Multiple Comorbidity	1,423 (43.9%)	988 (71.4%)	435 (28.6%)		480 (33.0%)	943 (67.0%)		550 (40.0%)	438 (31.5%)	206 (13.8%)	229 (14.8%)	
Mental health Depression & Anxiety												
No or Mild	3008	1876 (62.3%)	1132 (37.7%)	**0.002**	1224 (41.2%)	1784 (58.9%)	**0.010**	925 (31.2%)	951 (31.1%)	427 (14.0%)	705 (23.6%)	**0.019**
Moderate or Severe	362	266 (75.2%)	96 (24.8%)		106 (29.8%)	256 (70.2%)		161 (39.8%)	105 (35.5%)	49 (12.6%)	47 (12.2%)	

Weekly MVPA minutes mean 152.3; sd: 269.0. Daily sedentary hours mean:6.5; sd: 3.6. Row percentages were shown in this table P-values in bold indicate statistical significance (p < 0.05).

### Social factors associated with MVPA time, sedentary time, and activity pattern groups

#### Social factors associated with MVPA and sedentary time

Table 2 shows factors associated with MVPA time and sedentary time using multivariate linear regression. Older adults who were female (b=−55.79, 95% Confidence Interval [CI]: −101.5, −10.52), aged 75 years and above (b=−51.47, 95% CI:−98.90, −4.04) had significantly less weekly MVPA time compared to their counterparts. Older adults who had an annual household income of over 75,000 US Dollars (b = 60.90, 95% CI: 3.98, 117.81) had significantly more weekly MVPA time compared to those with lower annual household income. Non-Hispanic Black older adults had significantly fewer daily sedentary time (hours) than Non-Hispanic Whites (b: −0.63, 95% CI: −1.18, −0.08, *p* = 0.026). Similarly, Hispanic, Asian, and other ethnic groups exhibited significantly lower sedentary hours (b: −0.72, 95% CI: −1.31, −0.13, *p* = 0.017).

**Table 2:** Associations between Social Factors and Physical Activity Patterns

Social Factors	Weekly MVPA Minutes *n* = 2430		Daily Sedentary Hours *n* = 2218		High MVPA time Low sedentary time		High MVPA time High sedentary time		Low MVPA time Low sedentary time	
	Coef	95% CI	Coef	95% CI	AOR	95% CI	AOR	95% CI	AOR	95% CI
Sex										
Male (ref)										
Female	−55.79*	−101.05, −10.52	−0.43	−0.90, 0.04	0.85	0.57, 1.26	0.78	0.48, 1.28	1.22	0.86, 1.73
Age Group										
65–74 (ref)										
75 and older	−51.47*	−98.90, −4.04	0.12	−0.35, 0.59	0.69	0.46, 1.02	0.81	0.51, 1.30	1.15	0.82, 1.61
Race/Ethnicity										
Non-Hispanic White (ref)										
Non-Hispanic Black	−31.20	−6.81, 68.47	−0.63*	−1.18, −0.08	0.89	0.44, 1.80	0.96	0.44, 2.11	1.18	0.72, 1.93
Hispanic, Asian, and Others	23.23	3.98, 117.82	−0.72*	−1.31, −0.13	1.27	0.79, 2.03	1.35	0.72, 2.56	2.06**	1.35, 3.13
Annual Household Income (US Dollars)										
Low: <35,000 (ref)										
Middle: 35,000 to 75,000	30.83	−6.81, 68.47	−0.58	−1.25, 0.09	1.77**	1.17, 2.66	0.95	0.52, 1.71	1.14	0.75, 1.73
High: >75,000	60.90*	3.98, 117.81	−0.42	−1.13, 0.28	2.16**	1.26, 3.68	1.68	0.85, 3.31	1.26	0.78, 2.01
Education										
High school or less (ref)										
Some college	21.60	−19.52, 62.72	0.33	−0.19, 0.86	1.23	0.76, 1.97	1.19	0.71, 1.97	1.03	0.76, 1.40
College or higher	14.37	−38.87, 67.60	0.14	−0.37, 0.64	1.65*	1.08, 2.53	1.71*	1.05, 2.78	1.29	0.92, 1.81

This table presents weighted associations between Social Factors and Physical Activity (MVPA time, Sedentary time, and Activity pattern groups)

All results were adjusted for sex, age group, race and ethnicity, annual household income, education, smoking status, marital status, BMI, comorbidity, and mental health condition

AOR: Adjusted Odds Ratio. Reference group: Low MVPA time & High sedentary time

* 0.01 < *p* < 0.05; ** 0.001 < *p* < 0.01; *** *p* < = 0.001

#### Social factors associated with activity pattern groups

Older adults with higher annual household income was significantly associated with increased odds of falling into the“High MVPA time & Low sedentary time”activity pattern groups, with middle-income (AOR: 1.77, 95% CI: 1.17, 2.66, *p* = 0.007) and high-income groups (AOR: 2.16, 95% CI: 1.26, 3.68, *p* = 0.005) showing higher odds of falling in this class compared with the reference group (“Low MVPA time & High sedentary time”). Individuals with college or higher education had higher odds of falling into the “High MVPA time & Low sedentary time” (AOR: 1.65, 95% CI: 1.08, 2.53, *p* = 0.022) and “High MVPA time & High sedentary time” (AOR: 1.71, 95% CI: 1.05, 2.78, *p* = 0.033) groups. Compared with the reference group, older adults who were Hispanic, Asian, and other ethnic groups had significantly higher odds of falling into the group “Low MVPA time & Low sedentary time” compared to Non-Hispanic Whites (AOR: 2.06, 95% CI: 1.35, 3.13, *p* = 0.001).

### Associations between frequent WAT use and PA patterns

Table 3 shows the associations between WAT use frequency and older adults’ physical activity adjusting for covariates. We found no significant differences in sedentary time were observed in frequent WAT use, infrequent WAT use or no WAT use among older adults. Older adults who frequently used WATs (i.e., using a WAT “Every day” or “Almost every day” in the past month) had significantly longer weekly MVPA times (b = 58.54, 95% CI: 4.73, 112.34), and had higher odds of falling in the activity pattern groups of “High MVPA time & Low sedentary time” (AOR = 1.92, 95% CI: 1.10, 3.34) compared to the reference group.

**Table 3:** Weighted adjusted associations between WAT Use pattern and physical activity patterns

WAT Use	Weekly MVPA Time (Minutes)		Daily Sedentary Time (Hours)		Activity Pattern Groups					
					High MVPA time Low sedentary time		High MVPA time High sedentary time		Low MVPA time Low sedentary time	
	Coefficient (95% CI)	*p*-value	Coefficient (95% CI)	*p*-value	AOR (95% CI)	*p*-value	AOR (95% CI)	*p*-value	AOR (95% CI)	*p*-value
No Use (ref)										
Infrequent Use	−36.52 (−79.17, 6.12)	0.572	0.47 (−0.46, 1.40)	0.314	0.92 (0.40, 2.13)	0.840	1.01 (0.31, 3.22)	0.992	0.95 (0.43, 2.10)	0.890
Frequent Use	58.54 (4.73, 112.34)	**0.033**	−0.25 (−1.02, 0.52)	0.518	1.92 (1.10, 3.34)	**0.022**	1.63 (0.78, 3.42)	0.195	0.6718 (0.40, 1.14)	0.138

All results were adjusted for sex, age group, race and ethnicity, annual household income, education, smoking status, marital status, bmi, comorbidity, and mental health condition

*AOR *Adjusted Odds Ratio. Activity pattern groups reference group: Low MVPA time & High sedentary time P-values in bold indicate statistical significance (p < 0.05).

### The potential mediating role of WAT use on the associations between SES and PA patterns

Table 4 demonstrates a restricted indirect connection between SES and PA patterns. The results showed a minor indirect influence of income on MVPA time through WAT use (IE: 4.08, 95% CI: 0.04, 8.13), but no meaningful mediation for education or sedentary behavior was observed.

**Table 4:** Mediation analysis: adjusted direct and indirect association with MVPA and sedentary time

Weekly MVPA Time				Daily Sedentary Time			
Variables	Coefficients	95% CI	*p*	Variables	Coefficients	95% CI	*p*
*With Income via WAT use frequency*				*With Income via WAT use frequency*			
Total Effect	19.27	−4.17, 42.70	0.106	Total Effect	−0.39	− 0.04, 0.48	0.093
Indirect Effect	4.08	0.04, 8.13	0.048	Indirect Effect	−0.01	−0.05, 0.05	0.746
Direct Effect	15.18	0.10, 0.22	0.000	Direct Effect	−0.38	− 0.03, 0.49	0.082
Mediated Proportion	21%			No Mediation			
*With Education via WAT use frequency*				*With Education via WAT use frequency*			
Total Effect	19.48	−1.89, 40.85	0.074	Total Effect	−0.19	−0.07, 0.44	0.157
Indirect Effect	1.32	−0.57, 3.22	0.170	Indirect Effect	−0.00	−0.02, 0.02	0.743
Direct Effect	18.15	−3.33, 39.64	0.097	Direct Effect	0.19	−0.07, 0.45	0.151
Mediated Proportion	No Mediation			No Mediation			

## Discussion

The present study described U.S. older adults’ PA patterns and illustrated the associations among social factors, WAT use, and PA patterns. We found that over half of study sample reported less weekly MVPA time than the WHO recommendation (at least 150 min of moderate-intensity physical activity, and daily sedentary time more than 6 h). Sex, age, and income were significantly associated with MVPA time, while race and ethnicity were significantly associated with sedentary time. We also learned that frequent use of WATs was associated with more MVPA but not less sedentary behavior.

Consistent with previous literature, sex, age, and income were significantly associated with older adults’ MVPA time. Specifically, males generally engage in more physical activity than females (Azevedo et al., 2007; Hamrani et al., 2015), and younger age was associated increased PA, likely reflecting age-related decline in physiological function (McPhee et al., 2016), and higher income was associated with higher PA. Current evidence on education and PA association is mixed, with some studies reported that positive associations [13, 32], while others report a negative association [1], and we found no association between education and MVPA.

In this study, we also examined social factors associated with older adults’ sedentary behavior and found that ethnic minority older adults (Non-Hispanic Black Americans, Hispanic, Asian and other race and ethnicity) were less sedentary than Non-Hispanic White older adults. There are currently mixed findings in the literature regarding race and ethnicity differences in sedentary behavior. For example, one study examining low-income African American and White adults in the Southeastern U.S. found minimal differences in sedentary behavior between the two groups, but more television reviewing time was reported by African American adults [7]. Whereas other studies reported a higher prevalence of sedentary behavior among racial/ethnic minority groups compared to Non-Hispanic Whites, particularly in adolescents [14] and in women [25]. This pattern may reflect cultural differences in the perception and reporting of sedentary behavior, as well as genuine differences in daily routines, such as occupations, caregiving, and housework that require more frequent movements and physical activity. Additionally, cultural differences in transportation mode (public transit vs. private vehicles) may contribute to these variations. Given the limited and inconsistent evidence regarding racial and ethnic differences in sedentary behavior among U.S. older adults, further research is warranted, with a particular emphasis on ethnic minority populations.

In addition to investigating social factors associated with MVPA and sedentary behavior, this study created four activity pattern groups to capture older adults’ PA and sedentary time synergistically. Notably, older adults with intermediate or high annual household income were more likely to belong to the “High MVPA & Low Sedentary time” class compared to the reference group (“Low MVPA & High Sedentary time”). Previous studies with similar findings have suggested that higher income affords more resources to engage in physical activity and allows more time to engage in activities that reduce sedentary hours [2, 8, 32]. We observed a similar pattern among older adults with college degree or higher in the HINTS sample, consistent with literature that identified education as an important predictor of both MVPA and sedentary behavior [13, 24, 32].

The present study is among the first to illustrate associations between the frequency of WAT use and older adults’ self-reported PA patterns. While previous literature has largely focused on determinants of long-term use and adherence to WATs among older adults [10, 21, 23], limited attention has been given to WAT use patterns or frequency in the general adult population [4] and specifically among aging adults [17]. Given that only 65.5% of older adult WAT users reported frequent use (daily or almost daily) in 2019 and 2020 [17], it was especially important to examine how WAT use frequency may influence PA patterns. The findings of this research highlight the value of frequent WAT use in relation to PA. Importantly, frequent WAT use was found to partially explain the association between income and weekly MVPA time, supporting the study’s hypothesis. This suggests that older adults with higher annual household income may be more likely to engage in regular WAT use, which was also associated with their PA levels. Interestingly, this explanatory pathway was not observed in the association between income and sedentary behavior, possibly due to the design of WATs, which tend to emphasize features like steps monitoring and caloric expenditures aimed at increasing MVPA, rather than reducing sedentary behavior. To the authors’ knowledge, few studies have examined how WAT use interacts with the association of SES and PA in older adults. The findings of this study suggest that WAT access may be an important factor through which SES is associated with PA in the aging population [9].

Several limitations should be noted. First, all data in HINTS were self-reported, which is subject to recall bias and social desirability bias. Moreover, WAT use frequency and physical activity outcomes were both self-reported, raising the possibility of common method bias, which may inflate observed associations. Second, the cross-sectional design limited the power of the mediation analysis and precludes causal inference between the exposure, mediator, and outcome. As a result, the mediation analysis should be interpreted as exploratory given the study design. Third, WAT use frequency was derived from two questionnaire items assessing use in the past month, which does not capture prolonged or long-term WAT use behavior. Additionally, as the study data were collected before and during the first wave of the COVID-19 pandemic, changes in daily routines, mobility, and technology use during this period may have affected PA, sedentary behavior, and WAT use patterns among older adults. For COVID-19 pandemic related results, please refer to our previous publication . Last, we acknowledge that the MEDSEM command in Stata does not natively incorporate replicate weights for complex survey design. This is a methodological limitation of our analysis. Given the constraint that no readily available Stata procedure combines MEDSEM with HINTS-style replicate weight, we proceeded with the unweighted analysis.

Despite the limitations, the findings of the present study contribute to scientific knowledge and provide a foundation for future research, policy discussions, and clinical considerations regarding WATs and physical activity patterns in older adults. First, inequities in PA patterns were observed among older adults with different social backgrounds, highlighting the need for future research to examine how social determinants contribute to PA disparities. Second, this research generates hypotheses about the potential role of WAT use in supporting PA among older adults, particularly regarding the importance of consistent and frequent use. Considering the fact that physically more active older adults may be more likely to adopt and frequently use WATs, rather than WAT use leading to increased PA. Future studies with longitudinal designs are needed to disentangle the directionality of these associations and to determine whether WATs can effectively support sustained PA over time. Future research should also explore whether WATs may inadvertently become another digital determinant of health if access remains unequal (Sieck et al., 2021).

## Conclusion

This present study found that U.S. older adults reported overall low physical activity. Frequent WAT use was significantly associated with higher weekly MVPA PA patterns but was not significantly associated with sedentary behavior. Social disparities existed in PA patterns, and WAT use frequency partially explained the associations between income and weekly MVPA time. These findings suggest that frequent WAT use may be related to higher MVPA among older adults, particularly those with higher income. However, the cross-sectional design limits causal inference, and future longitudinal and intervention studies are needed to determine whether WATs can effectively support PA in older adults, including those from lower socioeconomic backgrounds.

## Data Availability

HINTS data are available publicly at the following URL: (https://hints.cancer.gov/data/download-data.aspx).
